# Kidney Stone Dissolution By Tetherless, Enzyme‐Loaded, Soft Magnetic Miniature Robots

**DOI:** 10.1002/adhm.202403423

**Published:** 2025-07-01

**Authors:** Afarin Khabbazian, Lauren Kwong, Aaron Lewis, Erica Liu, Noura Abdelrazec, Anna C. Bakenecker, Nil Fontanals, Guillem Lopez, Samuel Sánchez, Juan Manuel Lopez, Brian Carrillo, Monica Farcas, Chris Kallweit, Alfred C. H. Yu, Mir Behrad Khamesee, Veronika Magdanz

**Affiliations:** ^1^ Department of Mechanical Engineering University of Waterloo Waterloo N2L 3G1 Canada; ^2^ Medical Microrobotics Lab Department of Systems Design Engineering University of Waterloo Waterloo N2L 3G1 Canada; ^3^ Institute for Bioengineering of Catalonia Barcelona 08028 Spain; ^4^ Medical Engineering Technical University of Darmstadt Merckstr. 25 64283 Darmstadt Germany; ^5^ Catalan Institute for Research and Advanced Studies ICREA Barcelona 08028 Spain; ^6^ Department of Urology University of Barcelona Clinic Hospital Barcelona 08036 Spain; ^7^ Farcas Lab, Department of Surgery, Division of Urology, St. Michael's Hospital University of Toronto Toronto M5B 1W8 Canada; ^8^ Schlegel Research Institute for Aging and Department of Electrical and Computer Engineering University of Waterloo Ontario N2J0E2 Canada; ^9^ Waterloo Institute for Nanotechnology Waterloo N2L 3G1 Canada

**Keywords:** enzymatic, kidney stones, magnetic, microrobots, renal stones, soft robots

## Abstract

Kidney stones are some of the most common urinary diseases, affecting 12% of the population. The high prevalence and recurrence of this disease urges the development of more targeted and effective treatment with lower side effects and less invasiveness avoiding recurrence and prolonged drug administration. Particularly for recurring stone formers, the current methods of persistent drug treatment and repetitive surgeries for stone removal are unsatisfying solutions that bring a huge burden to the patients and healthcare systems. For these reasons, a delivery strategy that provides drug administration at the disease site through active, wireless transport is urgently needed to improve urinary tract disease treatment. A wireless treatment of kidney stones is proposed with the help of flexible, magnetically steered, enzymatically active robots. These robots are designed to navigate in the urinary tract and locally dissolve the stones by action of embedded urease. The robots are made of millimeter‐sized, gelatin‐based polymer strips with embedded micromagnets and encapsulated urease which constantly converts urea to increase urinary pH. This study demonstrates enhanced stone dissolution and the robots' magnetic navigation through the different parts of a 3D printed human urinary tract model. A clinical ultrasound system allows real‐time localization of the robots. This research proposes a less invasive and more targeted strategy for medical interventions in the urinary tract with potential to circumvent surgery in case of uric acid kidney stones, which is relevant especially in the light of high prevalence and recurrence of kidney stones.

## Graphical abstract

Wireless magnetic robots are developed for kidney stone dissolution. The robots navigate through a urinary tract model from bladder and ureter to renal pelvis with the help of external magnetic fields. The filaments are loaded with urease enzyme which catalyzes urea to carbon dioxide and ammonia, thereby increasing pH to basic regime and supporting dissolution of uric acid kidney stones.

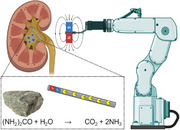

## 1 Introduction to Miniature Robots for Noninvasive Medical Tasks

The vision of non‐invasive medical devices is starting to come true thanks to the advances in nanotechnology, imaging, material engineering and technology. Over the last two decades, many approaches to developing wireless microrobots have been proposed. Microrobots are defined as microscale, actively moving wireless devices that are performing desired tasks under remote control. Their propulsion mechanisms include physical (magnetic, acoustic, light, electric), chemical or biological power sources that enable their forward motion and precise directional control.^[^ ^1^, ^2^, ^3^, ^4^ ^]^ The main potential of micro‐and nanorobots lies in the medical field, promising to make drug and cell delivery much more targeted by active transport mechanisms. For this reason, some of the most exciting advances have been in targeted bladder cancer treatment by urease‐driven, drug‐loaded nanomotors,^[^ ^5^, ^6^, ^7^ ^]^ drug delivery in the gastrointestinal tract to overcome poor absorption of oral drugs,^[^ ^8^ ^]^ and for stem cell delivery in the brain.^[^ ^9^ ^]^ Flexibility of microrobots offers specific benefits such as gentle interaction with surroundings and the ability to squeeze through tight spaces by changing their shape.^[^ ^3^ ^]^ Further, the ability to interact with soft tissue and cells without mechanical damage is crucial for biomedical applications. The state‐of‐the‐art flexible magnetic microrobots include artificial and biohybrid approaches. An artificial robotic sperm was developed by electrospinning to create a flexible polystyrene tail with a magnetic head.^[^ ^10^ ^]^ This millimeter‐sized robotic sperm was actuated with oscillating magnetic fields and demonstrated controlled forward motion. Flexible artificial microrobots have also been developed by fabricating chains of paramagnetic particles,^[^ ^11^, ^12^ ^]^ magnetic rods connected with flexible hinges,^[^ ^13^ ^]^ hydrogel microrobots with programmable motility,^[^ ^14^ ^]^ polymer sheets with certain magnetization profiles^[^ ^15^ ^]^ or elastomeric jellyfish‐like robots.^[^ ^16^ ^]^ As a biohybrid approach to magnetic flagellated robots, sperm cells were coated with magnetic nanoparticles, and actuated by oscillating^[^ ^17^ ^]^ or rotating magnetic fields^[^ ^18^ ^]^. Lately, flexible small scale robots are starting to be designed for their translation into clinical applications such as noninvasive surgery, cell manipulation and drug delivery. The potential impact of such microrobots lies in the ability to perform wireless actuation, reach remote sites and performance of therapeutic action. Overall, such medical microrobots aim to make medical interventions less invasive and more targeted. The optimum performance of such robots can only be achieved by tailoring the design and control mechanisms to the particular medical application and intended physiological environment. For this reason, recently, magnetic tetherless robots have been developed to target specific medical applications such as blood clot removal^[^ ^19^, ^20^, ^21^ ^]^, the treatment of aneurysms,^[^ ^22^ ^]^ biopsy,^[^ ^23^ ^]^ gastric sampling,^[^ ^24^ ^]^ cell manipulation,^[^ ^25^ ^]^ and drug or cell delivery.^[^ ^26^, ^27^, ^28^ ^]^ Current challenges toward further advances of microrobots toward clinical translation include the specific tailoring of the robots, as well as imaging and control systems to fulfill the requirements of the specific application and target site. In the here presented study, we propose a new field of application of flexible magnetic small scale robots, by demonstrating the navigation and therapeutic action of enzyme‐loaded hydrogel robots for the wireless dissolution of kidney stones in a human urinary tract model.

### 1.1 Kidney Stone Disease and Treatment

The prevalence of kidney stones in the world‐wide population is around 12%, and constantly rising, mostly due to increased obesity, diabetes type 2 as well as a diet high in salt and carbonated drinks. Once a person suffers from a kidney stone, there is a 70–80% chance of recurring stones.^[^ ^29^ ^]^ Overall, kidney stones can cause a reduction in quality of life and increased risk of chronic kidney disease. Kidney failure or infection are potential severe consequences of kidney stone disease. Kidney stones are crystals formed from calcium oxalate, calcium phosphate, uric acid, struvite, or cystine. Around 13% of kidney stones predominantly consist of uric acid.^[^ ^30^ ^]^ Symptomatic kidney stones are usually located in the ureter or uteropelvic junction and cause pain due to ureter blockage. Common treatment involves chemolysis by oral administration of pH increasing chemicals (citrate or bicarbonate). A low urine pH is strongly related to uric acid stone formation. It is known that the dissolution of uric acid stones is greatly increased at a pH higher than 6. Novel techniques for the dissolution of uric acid kidney stones have been proposed with the help of basifying agents such as N ‐acetyl‐ cysteine or theobromine. These agents hold promise in oral therapy in addition to citrate or bicarbonate.^[^ ^31^ ^]^ Rarely, direct irrigation with a catheter is used for local chemolysis of the stones. Possible chemolytic drugs include theobromine, N‐acetylcysteine or Hemiacidrin, but downsides of catheter‐based chemolysis if the danger of infection or sepsis during the constant irrigation process and long hospitalization of the patient (several weeks).^[^ ^32^, ^33^, ^34^ ^]^ In case of severe pain, blockage of the urine outflow or other urgent situations, surgery is performed to remove the kidney stones or break them down. Surgical techniques include shockwave lithotripsy, endoscopy, laparoscopy or open surgery. Surgical management is the cornerstone of kidney stone treatment, but high recurrence rates and associated complications necessitate the existence of alternative medical treatment options, aiming at dissolution of kidney stones and prevention of recurrence.^[^ ^35^ ^]^

### 1.2 Kidney Stone Dissolution by Soft Magnetic Robots

**Figure 1:**
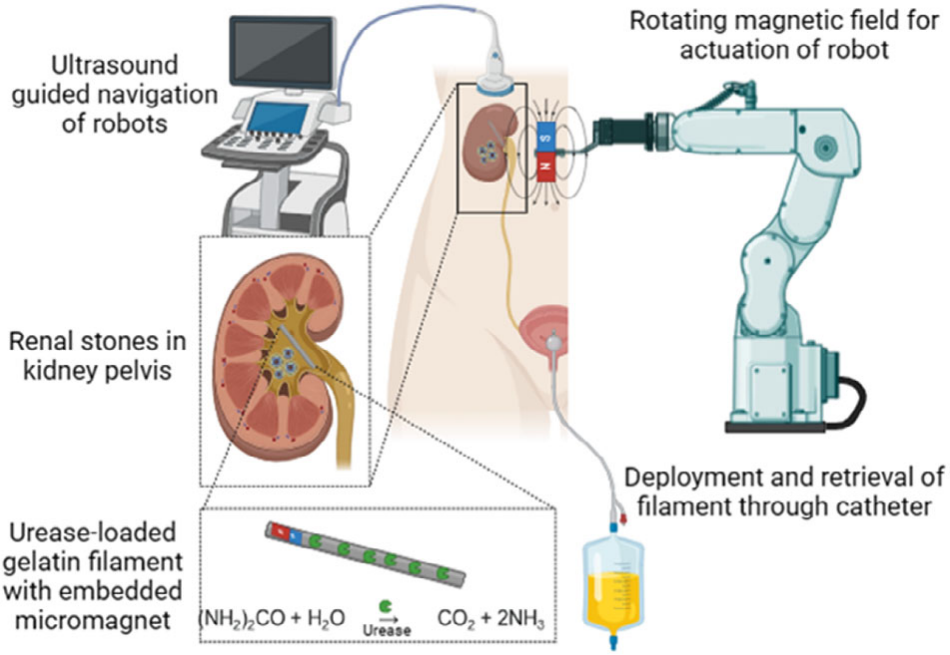
Concept of minimally invasive kidney stone dissolution by enzyme‐loaded magnetic small scale robots illustrating the potential future impact. Minimally invasive deployment of enzyme‐loaded robots through foley catheter into bladder or ureter and magnetic navigation to kidney stone location under ultrasound guidance.

In this study, we explore a new application of magnetic soft miniature robots in medicine, particularly for the noninvasive dissolution of kidney stones. We demonstrate the enhanced stone chemolysis by local delivery of an urease‐loaded magnetic, flexible gelatin filament to real human kidney stones. The urinary pH is constantly increased by the action of the encapsulated urease, thereby facilitating the accelerated dissolution of renal calculi in synthetic human urine. We demonstrate the controlled actuation of the filaments and enzymatic activity over extended time periods. In addition, thanks to the embedded micromagnet, the filaments can easily be visualized by ultrasound. The objective of this study is to design such soft magnetic robots for kidney stone dissolution and test them in an in vitro setting, mimicking the in vivo environment in terms of body fluids and complex human organ anatomy as closely as possible. This aims at evaluating the robot performance in terms of navigation, imaging and kidney stone dissolution and assess its suitability as tool in urology. **Figure** **1** illustrates the concept of this approach, aiming at introduction of the flexible filaments through catheters into the bladder or renal pelvis with a minimal invasive technique. A magnetic control setup compatible with a clinical setting provides an external rotating magnetic field which allows the actuation and navigation of the robots inside the urinary tract, and placement close to the kidney stone for most effective localized treatment. We demonstrate a successful, consistent increase of urine pH over up to 3 months by the same filament, and significantly enhanced kidney stone dissolution within five days. Noninvasive treatment can be envisioned by placement of the filaments inside the renal pelvis and external wireless fixation by an external magnetic patch placed on the skin. This could facilitate noninvasive local action of the robots for several days, potentially circumventing catheterization, hospitalization, and potential risks of infection.

## 2 Results

### 2.1 Fabrication and Characterization of Soft Magnetic Filaments

**Figure 2:**
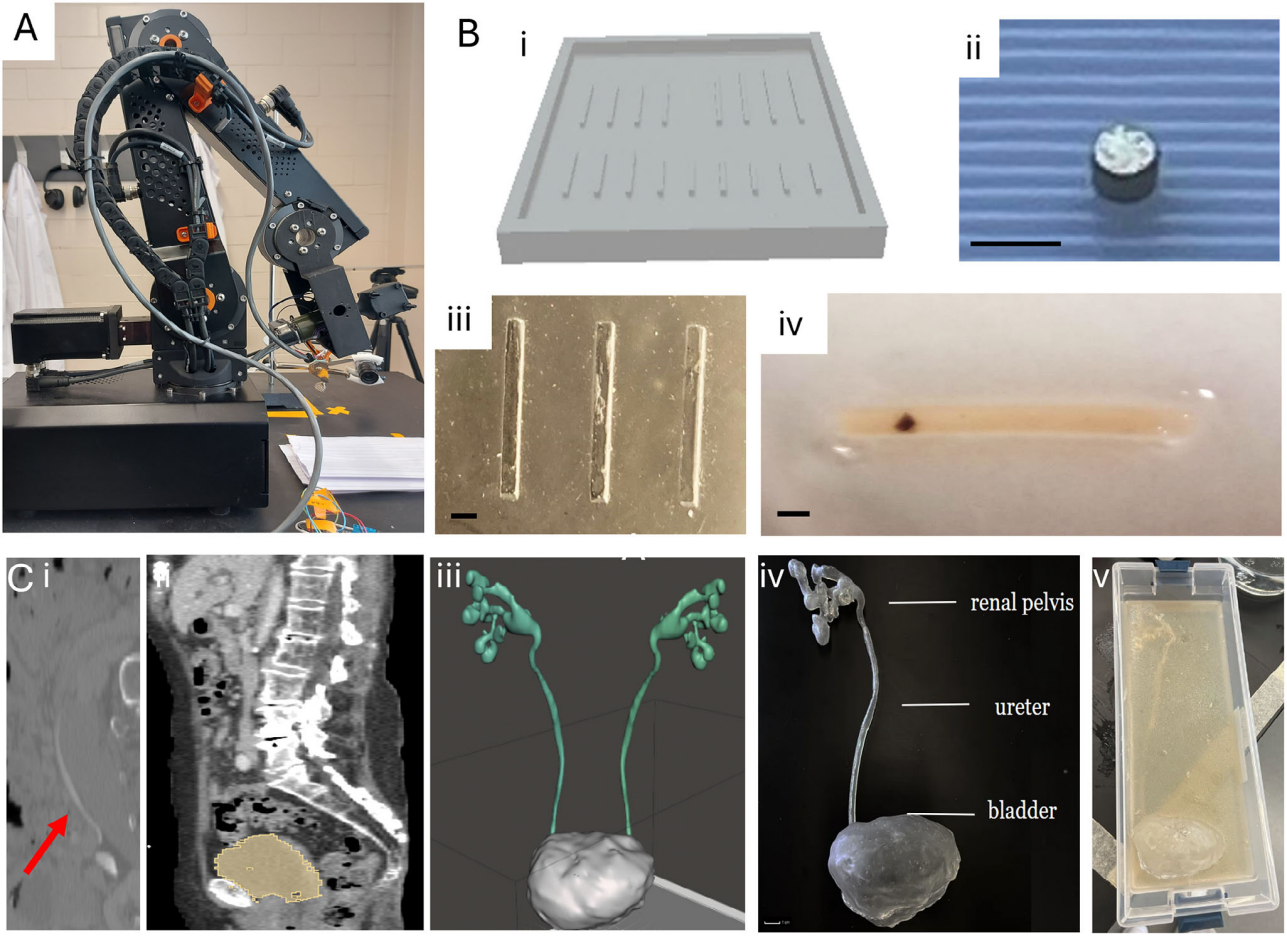
Magnetic actuation setup and fabrication of flexible filaments. A) Magnetic control system consisting of a 4 degree of freedom robot arm steering a DC motor that rotated a casing containing a permanent magnet. Bi) 3D printed negative mold cast for fabrication of gelatin filaments. Bii) 0.5–0.7 mm micromagnet. Biii) positive PDMS mold for UV crosslinking of gelatin methacrylate with embedded micromagnet Biv) resulting soft filament with embedded micromagnet and loaded enzyme urease. C) Fabrication of human anatomical urinary tract model by segmentation and selection of organ of interest from CT images of ureter (Ci, red arrow pointing at ureter) and bladder (Cii), used to reconstruct the urinary tract including bladder, ureter and renal pelvis (Ciii). (Civ) 3D printed transparent elastic model, (Cv) embedded for ultrasound imaging. Scale bars are 1 mm.

**Figure 3:**
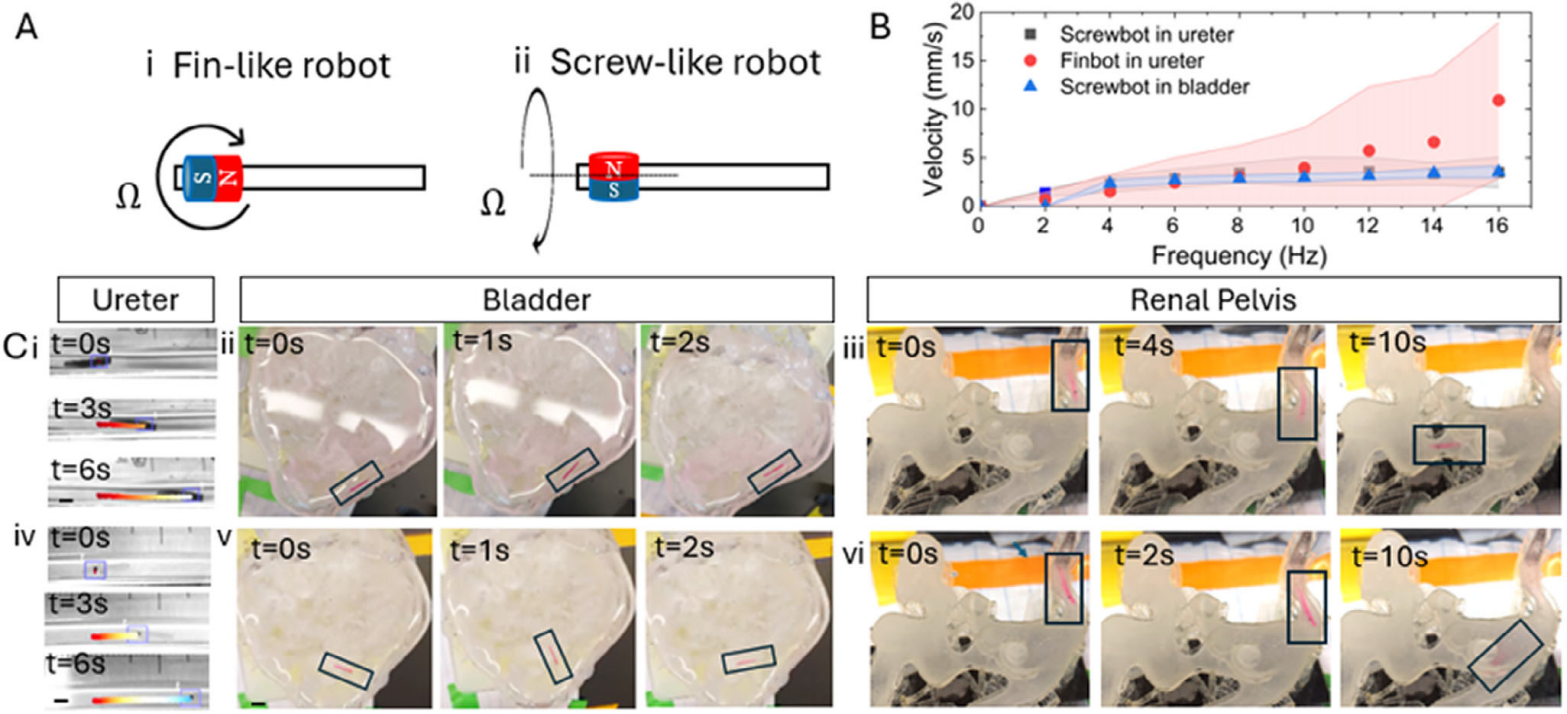
Two configurations of soft magnetic filaments are actuated in bladder, ureter and renal pelvis. Ai) Torque‐driven fin‐like robot with magnetization parallel to robot long axis (see Videos S1, S3, and S5, Supporting Information) and Aii) Force‐driven screw‐like robot with magnetization perpendicular to robot long axis (Video S2, S4, and S6, Supporting Information). B) Frequency response and resulting average robot velocities of screwbot and finbots in ureter, and screwbot in bladder. The shading corresponds to the standard deviation. Sample size n=10 robots for each data set. C) Image series of magnetic actuation of fin‐like robot in ureter (Ci, Video S1, Supporting Information), bladder model (Cii, Video S3, Supporting Information) and renal pelvis (Ciii, Video S5, Supporting Information), respectively. Image series of magnetic actuation and navigation of screw‐like robots in ureter (Civ, Video S2, Supporting Information), bladder (Cv, Video S4, Supporting Information) and inside the renal pelvis model (Cvi, Video S6, Supporting Information). Scale bars are 3 mm.

The flexible filaments were fabricated by molds, which were designed to yield 1x1x12 mm filaments that are envisioned to move easily through bladder, ureter and renal pelvis of a human, which smallest cavity width of 3–4 mm. A master mold was 3D printed (Figure 2 Bi), serving as a negative mold for a polydimethylsiloxane (PDMS) mold (see **Figure** **2**Biii). The PDMS mold was prepared using a 10:1 volume ratio of silicone elastomer base to curing agent. Hydrogel filaments were synthesized using 10% gelatin methacryloyl (GelMa) and Lithium phenyl‐2,4,6‐trimethylbenzoylphosphinate photo‐initiator (LAP, 4 mg mL^−1^), of which 20 µL were pipetted into the 1x1x12mm molds. Filaments were loaded with different concentrations of 15,000‐50,000 U/g urease by mixing the enzyme powder with the GelMa. Micromagnets (Zigmyster Magnets, 0.7 mm × 0.5 mm Ni‐coated NdFeB, Figure 2 Bii) were immersed into the GelMa on the front end of the mold (Figure 2 Biii). Gelma was then cross‐linked by exposure to UV light (Figure 2 Biv), which led to fixation of the micromagnet within the polymer network and encapsulation of the enzyme. To assess frequency response in the bladder, ureter and renal pelvis models two types of magnetic microrobots were fabricated by orienting the micromagnets in two possible ways: 1) perpendicular to the motorized actuator magnet to initiate fin‐like motion (**Figure** **3Ai**) or 2) parallel to initiate screw‐like motion (**Figure** **3**Aii). The actuation system containing a rotating permanent magnet driven by a DC motor and held by a robot arm is displayed in Figure 2A.

### 2.2 3D Printed Human Urinary Tract Model as Testing Environment

We demonstrate the controlled motion of the magnetic GelMa filaments in the anatomical environment of the urinary tract by actuation experiments in a 3D printed human urinary tract model (Figure 2C). This 3D printed organ model was designed based on the anatomical features obtained from medical images (Figure 2 Ci+ii), and reconstructed into a three‐dimensional design that was printed from flexible, transparent resin (Figure 2 and FigureCiv). This model was utilized to demonstrate how different motion configurations (fin‐like versus screw‐like robots, see Figure 3A) propel the magnetic filaments in the bulk fluid in the bladder, compared to the confinement of the ureter. Motion control is established with a rotating permanent magnet controlled on a robotic (Figure 2A). Details on the fabrication procedure and control setup can be found in the experimental section.

### 2.3 Magnetic Actuation of Urease‐Loaded Soft Magnetic Filaments

The drug‐loaded filaments have a permanent micromagnet placed inside, which facilitates untethered locomotion. It is known that a permanent magnet placed in magnetic field ($\vec{B}$) experiences the following torque and force,

(1) $$\vec{F}=V_{m}\left(\vec{M}\cdot \nabla \right)\vec{B}$$

(2) $$\vec{T}=V_{m}\left(\vec{M}\times \vec{B}\right)$$

where Equation (1) describes the force ($\vec{F}$) exerted on a magnet due to the magnetic field gradient ($\nabla \vec{B}$) and Equation (2) represents the torque ($\vec{T}$) experienced by a magnet in a magnetic field ($\vec{B}$). In these equations *V*_*m*_ represents the volume of the magnet and $\vec{M}$ is its magnetization vector. The details of the customized magnetic actuation setup was described in a previous work.^[^ ^36^ ^]^ The magnetic field generated by the permanent magnet exerts a magnetic field strength of 7 mT at a distance of 5cm, which was the standard working distance for the experiments. If the filament is placed in the magnetic field so that the magnetization vector is parallel to the field lines, the main component acting on the filament is the force and in the case of a rotating field, the filament follows the motion of the external permanent magnet, aligning its magnetic field with the external rotating field), resulting in a screw like motion. This rotation of the filament is what makes the propulsion happen.

On the other hand, if the robot magnet's magnetization vector is perpendicular to the external field, the torque component acts greatly on the robot. In this case, the robot will try to align itself with the external actuator, which has a perpendicular magnetic field orientation. The filament is not able to align itself to the external field due to the confinement inside the tube. In this confined space, friction with the walls is generated and helps the robot move forward in a fin‐like back‐and forth motion.

When actuating the magnetic filaments with the two configurations through the bladder model, we observe 2 distinct behaviors. The screw‐like robots move fairly linear along the bladder wall (Video S4, Supporting Information), while the fin‐like filaments show a spinning motion with no clear forward motion (Figure 3 Cv, Video S3, Supporting Information). It demonstrates that the fin‐like filaments do not perform well in bulk fluids. Therefore, no frequency response characterization was performed for fin‐like robots in the bladder. Our frequency response experiments in the ureter (a tube with 4 mm diameter) demonstrate that the fin‐like filament velocity can be increased with increasing magnet rotational frequency to a maximum speed of 5 mm/s and levels out at frequencies above 8 Hz (Figure 3B; Video S1, Supporting Information). Further increase does not result in enhanced filament velocity. Overall, the fin‐like robots move at faster speeds than screw‐like robots in the ureter. This is based on their motion mechanism that is supported by friction with the surrounding ureter walls. The interaction of the flexible robot with the walls leads to an enhanced forward motion, resulting in higher average velocities. Different micromagnet locations within the filament, and the effect on resulting robot velocities and bending motion were investigated previously, and did not seem to have any significant influence.^[^ ^36^ ^]^

The most intricate anatomy of the urinary tract is found in the renal pelvis (Figure 2C and Figure 3C). It is a branched cavity with high curvature. We demonstrate that both types of robots can be navigated in this complex environment (Figure 3 Ciii and 3vi; Videos S5 and S6, Supporting Information) thanks to the flexibility of the robots. We did not find that the length of the robot obstructed their motion and opted for keeping the robot dimension (1x1x12 mm) to allow sufficient enzyme loading and structural integrity of the filaments. The screw‐like filaments can be controlled more precisely inside the renal pelvis due to their screw‐like motion that does not depend on friction along walls. The fin‐like robots would occasionally get out of control due to their spinning motion when moving without confinement in the larger parts of the renal pelvic cavity. The robot's paths were very consistent, particularly in the ureter and bladder. In the complex structure of the renal pelvis, the robot paths were less predictable and reproducible due to the curvatures and cavities.

### 2.4 Kidney Stone Dissolution

The objective of this work is to demonstrate a new pathway for local uric acid kidney stone dissolution in a minimal invasive and highly targeted way, by the action of magnetic, soft enzymatic filaments tailored to move through the human urinary tract. Therefore, in the next step, the enhanced dissolution of kidney stones in synthetic human urine when exposed to the magnetic urease‐loaded enzymes is investigated. The underlying chemical mechanism of the dissolution is based on urease catalyzing urea, ubiquitously found in urine, to carbon dioxide and ammonia (Figure 1), thereby basifying the urinary pH. The reason for uric acid kidney stone formation is often too low urinary pH, and the established treatment strategy is oral treatment with basifying chemicals such as citrate.^[^ ^37^ ^]^ We adapt a similar approach, with the difference that the urine is basified locally in the vicinity to the kidney stone by enzymatically active filaments, which is expected to be much more effective.

#### 2.4.1 Targeted Increase of Urinary pH

**Figure 4:**
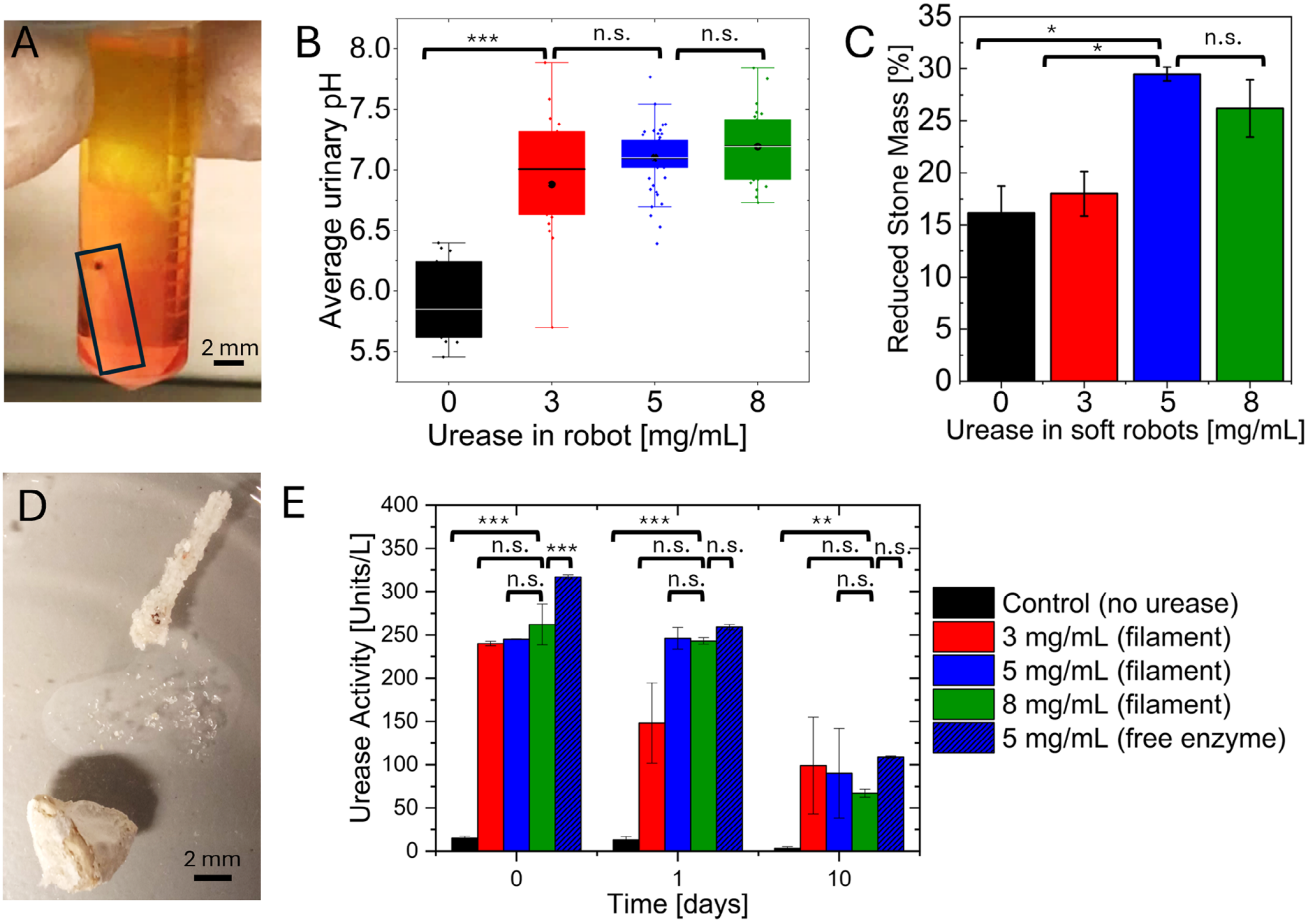
Kidney stone dissolution and urinary pH increase. A) The magnetic GelMa filament is actuated inside a vial with synthetic human urine with pH indicator. The box indicates the location of the transparent hydrogel filament with the black dot being the micromagnet. The pH indicator shows immediate pH change in the surroundings of the urease robot to more basic pH regime. B) Average urinary pH obtained after 24 hours of urease‐loaded filament exposure showing pH increase from pH 6 to pH 7.0 (3mg mL^−1^ urease), pH 7.1 (5mg mL^−1^) and pH 7.2 (8 mg mL^−1^ urease).Sample sizes were n=3 vials for each condition. C) Kidney stone weight reduction after 5 days exposure to soft robots with different amounts of urease loaded, indicating best stone reduction at 5 mg mL^−1^ urease inside robots. Sample sizes n=14 (0 mg mL^−1^ urease), n=23 (3 mg mL^−1^ urease), n=45 (5 mg mL^−1^ urease), n=17 (8 mg mL^−1^ urease) D) GelMa filament after incubation with kidney stones in synthetic urine for 5 days. Deposits can be observed on the filament surface, potentially from recrystallization of uric acid on its surface. E) Urease activity of encapsulated urease in filaments and free urease in synthetic human urine measured at 0, 1, and 10 days. The control is a filament without urease. Sample sizes are n=3 filaments per condition. Statistical analysis was done with one‐way ANOVA in Origin 2023b. See details of statistical analysis in Figure S4 (Supporting Information).

A low urine pH is strongly related to the uric acid stone formation.^[^ ^30^ ^]^ It is known that the dissolution of uric acid stones is greatly increased at a pH higher than 6, ideally in the regime of pH 7.0–7.2.^[^ ^38^ ^]^ Basifying agents such as citrate or bicarbonate react directly with acids in the urine to bring the pH to more basic regime, but are used up in the process. Thus, continuous oral administration of basifying agents are needed for extended periods of several months to achieve dissolution of uric acid stones. Our approach is based on a continuous, local enzymatic action by urease‐loaded filaments, which supports the catalysis of urea and thereby increases urinary pH to more basic condition. According to previous studies, a urine pH of 7.0–7.2 is contributing to chemolysis of uric acid stones and prevents further stone formation.^[^ ^30^ ^]^ First, the urinary pH increase by the urease‐loaded filaments was investigated. Figure S1 (Supporting Information) illustrates our first results investigating the pH increase of synthetic urine from pH 6 to pH 7 within 1 hour and up to pH 9 within 24 hours, of exposure to the urease‐loaded filaments with urease concentrations of 0–50 mg mL^−1^. The lowest concentration of 6 mg mL^−1^ urease was sufficient to increase the pH drastically within 24 hours, so we continued experiments with a range of 0–8 mg mL^−1^ urease. **Figure** **4**A shows the instant pH increase of the urine illustrated by the pH indicator phenol red added to the urine, which turns the solution from yellow to orange in the presence of the enzyme‐loaded filament, indicating a change from acidic to basic pH. Figure 4B illustrates that the pH increase to pH 7 within 24 hours was achieved with filaments containing 3‐8 mg mL^−1^ urease. The control sample contained a GelMa filament without urease. In this case, no overall pH change of the urine was detected and remained at pH 6. We also tested the long‐term enzymatic activity of the filaments (Figure S3, Supporting Information), and observed that the urease loaded inside the filaments is active for over 65 days. The filaments were immersed in 10 mL synthetic urine of pH 6, and after each 24 hours the pH was measured. Subsequently, the synthetic urine was removed and replaced with pH 6 fresh urine, and pH measured again after the next 24 hours. This was done for 65 days repeatedly. The synthetic urine which was used in the experiments is not a buffered solution, therefore, small fluctuations in pH are observed and can be considered natural, as they occur in real human urine. Further, slight changes in the enzymatic activity over the weeks, as well as slight changes in the synthetic urine, might have led to these fluctuations. A long term pH increase to above pH 7 could be obtained with the lowest urease concentration of 6 mg mL^−1^(Figure S3, Supporting Information). Enzyme activity of the GelMa filaments were performed over 10 days, as the envisioned application would likely be for 1–2 weeks to support an accelerated stone dissolution by the filaments which are temporarily placed into the renal pelvis in a wireless way. The continuous pH change of the synthetic human urine over elongated time periods is proof that the urease enzyme is active, as this pH change does not occur in the control sample without enzyme (Figure 4B, C, and E). The encapsulated enzyme is highly active on day 0 and 1 with over 200 U/L, and also maintains sufficient activity after 10 days to cause a significant urine pH basification. In fact, our experiments show that the encapsulated enzyme maintains its activity better on day 1 in relation its initial activity on day 0 compared to the free enzyme. There is an increase in overall enzyme activity when encapsulation amount is increased from 3‐5mg mL^−1^, but no further increase when elevating to 8 mg mL^−1^ urease inside the GelMa. This might be due to buried active enzyme sites in the GelMa, as well as inhibited substrate diffusion with increasing hydrogel concentration. Thus, 5 mg mL^−1^ urease shows to be the optimum enzyme concentration, which is also in line with our results for the pH increase and stone dissolution at 5 mg mL^−1^ urease.

#### 2.4.2 Enhanced Dissolution of Human Kidney Stones

After successful urine pH increase, the dissolution of human kidney stones was investigated in in vitro experiments. Kidney stones were obtained from local clinics and analyzed with Fourier transform infrared spectroscopy, and stones with a majority of uric acid content were selected (see Experimental Section). These uric acid stones were weighed on analytical scales before each experiment. The stone dissolution was measured by stone weight reduction over time. Kidney stones were immersed in 10 mL synthetic urine at pH 6, simulating the conditions in a stone patient's urinary tract. The pH of urine was monitored throughout the experiment over 5 days. After completion of the experiment, the uric acid stones were dried and weighed. The stone weight reduction was calculated and plotted over urine pH to evaluate any correlation between pH and stone reduction. As shown in Figure S2 (Supporting Information), most efficient stone reduction occurred in a pH range of 6.5–7.5, which is in line with previously reported studies.^[^ ^30^ ^]^ A large variation was observed due to the difference in stone sizes (and thus surface area), and different stone compositions. When the pH was increased above 7.5 due to high enzymatic activity, no further enhanced stone dissolution was observed. The most optimum stone dissolution was achieved with urease filaments containing 5 mg mL^−1^ urease (Figure 4C), which led to an average stone weight reduction of 30% within 5 days, and an average urine pH of 7.0. Figure 4C summarizes the average stone mass reduction dependent on the urease concentration in the filaments, with error bars indicating the standard error of the mean. When no urease was loaded into the filaments, about 15% of stone mass reduction was observed, possibly due to the uric acid adsorption onto the GelMa filaments. Filaments with 3 mg mL^−1^ urease led to no significant stone reduction, although the urine pH was increased to 6.9 on average. 5mg mL^−1^ urease had the best outcome with 30% stone mass reduction and pH 7.0, significantly increased compared to 0 and 3mg mL^−1^ (p = 0.03). Further increase of urease cargo (8mg mL^−1^) led to pH of 7.2 and stone mass reduction of 25%, so no further significant increase in stone mass reduction (p = 0.5). It can be concluded from this study that the optimum pH for stone dissolution is very important and too high urease activity (above 5 mg mL^−1^) and thus too high urine pH had a contrary effect on stone dissolution. Figure 4D shows an urease filament and a kidney stone after 5 days incubation in urine. Crystallization on the filament can be observed, which are possible uric acid crystals that were dissolved from the stone and recrystallized on the filament. The robot filament with embedded micromagnet remained structurally intact and could be handled easily. Within the time frame of the dissolution experiments (10 days), no disintegration, deformation and only little degradation of the filaments were observed. Conventional orally prescribed basifying agents such as citrate and bicarbonate were also tested to observe their effectiveness in the local dissolution of the uric acid stones compared to urease. Citrate and bicarbonate were added to the vials containing the synthetic urine and kidney stones. Although a burst increase of pH was observed, no continuous pH increase was increased. We also embedded up to 2 g mL^−1^ bicarbonate into the 10% GelMa filaments to test the local pH increase and kidney stone dissolution from robotic delivery of bicarbonate. The stone weight reduction was not significantly increased compared to the control sample. The reason for this is that the bicarbonate is released from the GelMa filament rather quickly and after a short burst change in pH, the pH measurements showed no difference between the bicarbonate filaments and the control samples. Hence, bicarbonate addition only leads to a one‐time increase of pH. Since the urine was replaced every 24h, there was no further pH increase observed after 24h by the bicarbonate filaments, thereby proving as an ineffective approach for consistent pH change compared to the catalytic action of urease. Trials with filaments containing a combination of urease and theobromine showed no significant improvement in pH change or stone reduction, but might require further investigation, particularly under urine flow conditions.

We have initially demonstrated the stability of the GelMa filaments for over 2 months (see Supporting Information Figure S3), although it is not necessary for the final application. The filaments are designed in size and softness in a way that they can be flushed out through the urethra after treatment and removal of external magnetic field. Additionally, the degradation of gelatin methacrylate filaments could be achieved by hydrolysis and depends on the surrounding pH. In preliminary experiments, we have investigated the swelling and degradation of the gelatin methacrylate network in urine of different pH by measuring the filament dimensions over 10 days (Figure S5, Supporting Information). Our results show that the degradation is dependent on the surrounding pH. At acidic pH of 5, GelMa filaments dissolve by 11% in side after 10 days when using 10% GelMa. Basic pH of 8 accelerates the filament hydrolysis in pH 8 to 20% of the initial filament size by day 10. in neutral pH, a degradation of 19% was observed by day 10. In order to aim for specific degradation target time, further investigation is needed to determine the optimum desired clinical treatment time of the filaments. Also, the filament degradation time can easily be tuned by the concentration of hydrogel and crosslinking density of the hydrogel. Swelling of the hydrogel filaments occurs of up to 200% of the original size of the GelMa filaments once they are immersed in the synthetic urine within 24 hours. Largest level of swelling is observed at pH8 at 24h (200%), followed by pH7 (185%) and pH5 (144%), as shown in Figure S5 in supporting information.

**Figure 5:**
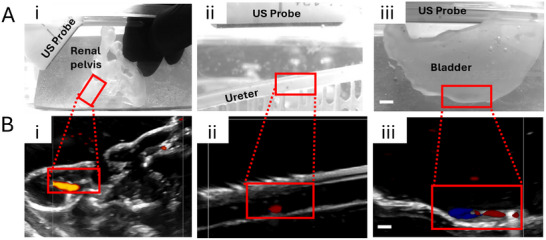
Ultrasound imaging of robots in real time. A) Optical images, and B) ultrasound images of magnetic filaments inside (i) renal pelvis, (ii) ureter and (iii) bladder phantom from simultaneous magnetic actuation and imaging. Red rectangles indicate location of the robots. Micromagnets give bright contrast in Doppler mode. Scale bars are 2 mm. See also Videos S7, S8, and S9 (Supporting Information).

Overall, the significant increase pH of urine over elongated periods strengthens the approach of using urease as enzyme instead of the release of basifying agents. The latter approach would require a daily replenishing of the drug inside the urine, because only limited amounts (e.g. a few milligrams of bicarbonate) can be loaded into the filaments. This approach would not support the approach of prolonged, noninvasive and consistent urinary pH increase.

### 2.5 Ultrasound Guided Localization and Motion Monitoring of Soft Magnetic Filaments in Organ Model

Proof‐of‐concept experiments were performed using a clinical ultrasound imaging system to demonstrate the compatibility of the filament robots and actuation system in a clinical imaging setup. Figure 2C illustrates the fabrication process of the human urinary tract model, starting with the reconstruction of a 3D anatomical model of the bladder, ureter and renal pelvis based on imaging data from computer tomography. (Figure 2 Ci+ii). The relevant organ was selected in each image slice (Figure 2 Cii) and reconstructed into a 3D model (Figure 2 Ciii). This design was then adjusted to be printable by stereolithography, resulting in a transparent, flexible real‐size organ model with anatomical features (Figure 2 Civ). This model was subsequently embedded in an ultrasound‐compatible agar / gelatin slab to serve as phantom tissue for ultrasound imaging (Figure 2 Cv). The model was then filled with water, and the flexible magnetic robots were introduced into the model organ. Thanks to the strong echogenicity of the neodymium micromagnet, the filament was easily detected by ultrasound imaging during magnetic navigation inside the organ phantoms (see **Figure** **5**B; Videos S7–S9, Supporting Information). The location of the robot and its motion in the presence of the rotating magnetic field can be visualized using the ultrasound scanner's color flow imaging mode that depicts bidirectional motion based on the phase shifts in ultrasound echoes returned from moving targets (Figure 5B; Videos S7–S9, Supporting Information). Video S7 (Supporting Information) illustrates the motion of the robot detected in the model in the lower bladder. The rotations of the filament in response to the magnetic actuation can be detected in the color flow imaging mode. This mode is usually utilized to visualize flow direction and velocity. Flow that travels away from the transducer is depicted in blue, and flow that is traveling toward the transducer is depicted in red. In Videos S7, S8, and S9 (Supporting Information) showing simultaneous magnetic actuation and imaging in the bladder, ureter and renal pelvis, a fast switching between red and blue colors can be observed. This signal stems from the micromagnet inside the filament serving as highly echogenic object, which is rotating constantly and creates this periodic color change. In fact, the frequency of the switching correlates to the applied magnetic rotational frequency of the actuator. Further, a generation of fluid turbulence by the robots can be observed in the surroundings of the robots in the ultrasound color flow image.

## 3 Conclusion

The wireless in situ chemolytic dissolution of uric acid kidney stones demonstrated in this study by the use of enzyme‐loaded flexible magnetic filaments is a new approach that potentially offers a nonsurgical method to remove kidney stones in the future and opens new pathways for other non‐invasive medical treatments in the kidney and urinary tract such as drug and cell delivery and removal of occlusions. The optimum urine basification to a pH of 7 was achieved throughout several weeks, supporting the enhanced local kidney stone dissolution. A uric acid kidney stone mass reduction of 30% was achieved within five days with 5 mg mL^−1^ urease‐loaded filaments, which shows a doubled dissolution rate in comparison to the control conditions. Note that complete stone dissolution is not needed, as smaller stones below the size of 4mm can be passed naturally.^[^ ^39^ ^]^ The gelatin filaments are envisioned to be retained in the renal pelvis by an external magnetic patch for as long as needed to continuously catalyze urine to increase urinary pH in a noninvasive manner for continuous dissolution of uric acid stones. The filaments with diameter of 1mm containing micromagnets are small enough to be passed through the ureter and bladder and urethra. Although the micromagnets are coated with nickel, toxicity and leaching studies should be conducted to investigate any release of toxic material from the micromagnets. Additional coating procedures could be applied to ensure the safety of the micromagnets for this application. In future studies, urine fluid flow should be integrated in the robot performance testing, as well as the ureter's peristaltic motion to mimic the ureter conditions more closely, before moving to in vivo studies. Loading of additional drugs, such as anti‐inflammatory, pain‐relief and antibiotic drugs could easily be implemented.

Together with the magnetic control system which applies weak magnetic fields, this will be a safe and noninvasive control and imaging system. With the help of the complete 3D printed urinary tract, the robot insertion and retrieval can also be tested in follow‐up experiments.

## 4 Experimental Section

### Materials

Chemicals were purchased from Millipore Sigma, except for PDMS (Sylgard 184, Ellsworth Adhesives). Micromagnets were obtained from Zigmyster Magnets, ON, Canada.

### Kidney Stone Analysis

Kidney stones were kindly donated by The Hospital Clinic Barcelona, as well as the Laboratory Reference Centre in Hamilton. Ethics approval to work with human kidney stones was obtained from the Research Ethics board of the University of Waterloo (#44982). The renal calculi were released completely anonymized so that there is no patient‐identifying information linked to the stones. The composition of the kidney stones was analyzed by Fourier transform spectroscopy (FTIR) spectroscopy. Stones with peaks at 1021 nm and 1638 nm were identified as containing a high amount of uric acid and hence were selected for this study.

### Synthetic Human Urine Preparation

Synthetic human urine medium was prepared based on the composition described by Sarigul et al.^[^ ^40^ ^]^ All required compounds were sourced from Millipore Sigma. The compounds were dissolved in MilliQ water at 60°C using a magnetic stirrer. After preparation, the medium was sterile filtered and adjusted to a pH of approximately 5.5 with hydrochloric acid to mimic the urinary conditions of a kidney stone patient. This was used as the liquid medium for the bladder, ureter and renal pelvis models, as well the local dissolution of kidney stones, wherein the urine was replaced daily and its pH was monitored over a four‐day period.

### Fabrication of Soft Magnetic Gelatin Filaments

The small scale magnetic robots were fabricated from lyophilized Gelatin Methacrylate Photogel (GelMa) (Cellink 5208) which is mixed with sterile water or phosphate‐buffered saline to make a final hydrogel of 10% GelMa which is then mixed with 4 mg mL^−1^ of photo initiator LAP (Lithium phenyl(2,4,6‐trimethylbenzoyl)phosphinate). To shape the filaments, a negative mold is 3D printed by Formlabs stereolithography and Grey resin (Figure 2Bi). The Polydimethylsiloxane (PDMS) mold was prepared using a 10:1 volume ratio of silicone elastomer base to curing agent and casting PDMS into the mold to obtain the actual filament mold with dimensions of 1x1x12mm channels. The filaments are crosslinked by adding the hydrogel solution to the channels in the molds (Figure 2 Biii), placing a permanent micromagnet (0.7 mm × 0.5 mm, Figure 2Bii) made of NdFeB grade 52 with Ni coating (Zigmyster Magnets, Toronto) inside the liquid hydrogel, and exposing the filaments to 405 nm UV light for 4 minutes, creating the final soft filament robot (Fig 2Biv).

### Fabrication of Enzyme‐Loaded Filaments

In order to create the enzyme‐loaded filaments, various concentrations of urease powder (15 000–50 000 U/mg, Millipore Sigma) sourced from Canavalia ensiformis were mixed into the GelMa solution prior to polymerization under UV light, with no micromagnets embedded. The urease concentrations used in the GelMa solution were 3 mg mL^−1^ (n = 23), 5 mg mL^−1^ (n = 45), and 8 mg mL^−1^ (n = 14). Controls were designated as GelMa filaments with no urease (n = 14). Each filament was placed into a test tube containing a single kidney stone (initial mass ranging from 0.0019 to 0.0541 g) and 10 mL of sterile‐filtered synthetic human urine, adjusted to pH 5.6 with 0.1 M HCl solution. After initial pH measurements were recorded using a pH meter, samples were placed on a rotating mixer to agitate continuously for 24 hours. Following this period, the pH of each test tube was recorded again and the synthetic urine medium was replaced. This process was repeated for four consecutive days. At the end of the experimental duration, the kidney stones were removed from the test tubes, dried in an incubator at 60°C for 72 hours, and their final masses were recorded. The percent change in kidney stone mass was calculated by comparing the initial and final masses.

### Frequency Response Experiments

To increase the visibility of the filaments for automatic tracking in the videos, they are soaked in rhodamine solution which dyes them red. The final shape is a 12mm long filament with a square cross section of 1 mm × 1 mm. The experimental setup for the magnetic actuation of the flexible filaments includes a robotic arm (igus Robolink) shown equipped with a DC motor (brushed 24 volt DC motor from maxon motor) attached to its end‐effector (Figure 2A). The motor facilitates rotational movement for a permanent magnet with dimensions $1^{\prime \prime }\;\text{diameter}\times {\frac{1}{2}}^{\prime \prime }\;\text{thickness}$ (25.4 mm × 12.7 mm) made of NdFeB, grade N52, where the surface field of the magnet is 5233 Gauss (0.5233 T), *B*_rmax_ is 14,800 Gauss (1.48 T) and *B*_Hmax_ is 52 MGOe (416 kJ/m^3^). The rotation speed can be controlled through Arduino Uno and a Motor Shield from Adafruit creating a range of frequency between 2 and 16 Hz. For each set of experiments the response of the filaments to the actuation was recorded using a camera from Basler. Then the video was processed using a python based algorithm (The Nano‐micromotor Tracking Tool (NMTT) https://github.com/rafamestre/NMTT‐nanomicromotor‐tracking‐tool), facilitating tracking of particles. The output of the code gives the total displacement of the robot, and having the duration of the movement, the response of the filaments to each frequency was established. The analysis of motion was performed by a similar Python‐based tool (Nano‐micromotor Analysis Tool (NMAT) https://github.com/rafamestre/NMAT‐nanomicromotor‐analysis‐tool), providing the average speed of the filaments based on data gathered from the NMTT.

### 3D Printing of Urinary Tract

The urinary tract model was segmented from real patient computed tomography (CT) scans derived from The Cancer Imaging Archive. The renal pelvis, ureter and bladder were segmented individually in Slicer 3D and refined and hollowed for printing in Meshmixer. The phantom models the urinary tract of an adult. The resulting renal pelvis model has a height of 41.30mm and a maximum major calyces length of 43.28mm. The segmented ureter has a diameter range of 2.54–5.14mm and a length of 123.3mm. The average length of an adult ureter is 280–320mm of an adult. This was accounted for by adding a lengthening silicon tube with an inner diameter of 5/16’’ to have a total length of 280mm. The bladder has a length, height, and diameter of 75.05, 59.10, and 65.73mm, respectively. The wall thickness for the renal pelvis and ureter is 0.40 and 3mm for the bladder. Printing involved the use of Elastic 50A resin on the Form3 printer. Following the printing process, a series of post‐processing steps were executed tailored to the segments. The renal pelvis and ureter required an ethanol wash of 20 minutes, ethanol flushing to eliminate any residual resin, an air dry of 30 minutes, UV cure of 33 minutes, and the removal of supports. The bladder was cleaved in half before printing to avoid cupping during printing. The bladder required a 10 minute ethanol wash, UV cure of 20 minutes, and removal of supports. The segments were then assembled and attached using Gorilla glue to ensure a waterproof seal between connections.

### Kidney Stone Dissolution Experiments

Kidney stone dissolution experiments were performed in vials containing 10 mL artificial urine at initial pH of 5.6 with one uric acid stone placed in each vial. Initial stone weights were measured with an analytical balance. The vials were placed on shakers for 24 hours. The pH was measured every day for 4 days, with the urine being renewed every 24 hours. After completion of experiments, the stones were taken out, dried in an oven at 40 degrees for 72 hours and the final dry weight was measured on an analytical balance.

### Urease Activity Assays

Enzymatic activity of urease‐loaded hydrogel filaments was compared with that of free urease in solution by the use of the Sigma Aldrich urease activity assay kit (product nr. MAK120). A stock solution of free urease (1 mg mL^−1^) was diluted using 10 mM assay buffer (pH 7.0) and added to clear 96 well plates to replicate the total urease content present in the hydrogel filaments, normalized to the filament volume of 12 mm^3^. The assay was performed according to the manufacturer's instructions, reading absorbance at 670nm with a Tecan spectrophotometric multiwell plate reader of the samples along with a series of ammonium standards.

### Ultrasound Imaging of Soft Filaments in Organ Model

Color flow imaging (6.3 MHz central frequency, 4.5 kHz pulse repetition frequency) was performed using a linear ultrasound probe (L4‐12t‐RS; GE HealthCare, Chicago, IL, USA) attached to a clinical ultrasound scanner (Venue Go; GE HealthCare, Chicago, IL, USA). For ultrasound imaging purposes, the 3D printed anatomical organ model was embedded in an ultrasound‐compatible agar/gelatin slab containing 1.5% agar, 3.75% gelatin and 0.3% potassium sorbate as preservative. The agar/gelatin mix was prepared by dissolving all reagents in 1 liter of distilled water and pouring into a plastic container containing the urinary tract model (Figure2Cv), and letting it set in the fridge overnight. Characterized in a previous study,^[^ ^41^, ^42^ ^]^ the elastic modulus, acoustic attenuation, and speed‐of‐sound of this agar/gelatin formulation are 35.9 kPa, 0.145 dB/($\mathrm{cm}\dot{rmMHz}$), and 1510 m/s respectively. These properties are similar to those of human soft tissue.

### Filament Swelling and Degradation Analysis

Three vials with each containing a GelMa filament and 10 mL synthetic urine were prepared and kept at pH 5,7 and 8 at 37 degrees Celsius for 10 days. The filament surface area was measured from pictures taken every day over 10 days in (mm^2^) using the polygon tracing tool on ImageJ.

### Statistical Analysis

Stone and filament sizes were normalized to their respective initial sizes on day 0. Data are presented as average ± Standard error of the mean. Significant differences were calculated by one‐way ANOVA with Bonferroni method and significance level of 0.05 in Origin 2023b. Average values, standard errors of the mean, p values, R square values and coefficient of variation are noted in supporting information Figure S4. Sample sizes are noted in each figure caption. *** means *p* < 0.001, ** means *p* < 0.01, * means *p* < 0.05, n.s. means no significant difference.

## Conflict of Interest

The authors declare no conflict of interest.

## Supporting information

Supporting Information

Supplemental Video 1

Supplemental Video 2

Supplemental Video 3

Supplemental Video 4

Supplemental Video 5

Supplemental Video 6

Supplemental Video 7

Supplemental Video 8

Supplemental Video 9

## Data Availability

The data that support the findings of this study are available from the corresponding author upon reasonable request.

## References

[1] B. J. Nelson , I. K. Kaliakatsos , J. J. Abbott , Annu. Rev. Biomed. Eng. 2010, 12, 55. 20415589 10.1146/annurev-bioeng-010510-103409

[2] H. Ceylan , J. Giltinan , K. Kozielski , M. Sitti , Mobile microrobots for bioengineering applications 2017. 10.1039/c7lc00064b 28480466

[3] M. Medina‐Sánchez , V. Magdanz , M. Guix , V. M. Fomin , O. G. Schmidt , Adv. Funct. Mater. 2018, 28, 1707228.

[4] V. Iacovacci , E. Diller , D. Ahmed , A. Menciassi , Annu. Rev. Biomed. Eng. 2024, 26, 561. 38594937 10.1146/annurev-bioeng-081523-033131

[5] X. Ma , X. Wang , K. Hahn , S. Sánchez , ACS Nano 2016, 10, 3597. 26863183 10.1021/acsnano.5b08067

[6] A. C. Hortelao , C. Simó , M. Guix , S. Guallar‐Garrido , E. Julián , D. Vilela , L. Rejc , P. Ramos‐Cabrer , U. Cossío , V. Gómez‐Vallejo , T. Patiño , J. Llop , S. Sánchez , Sci. Rob. 2021, 6, eabd2823. 10.1126/scirobotics.abd2823 34043548

[7] C. Simó , M. Serra‐Casablancas , A. C. Hortelao , V. Di Carlo , S. Guallar‐Garrido , S. Plaza‐García , R. M. Rabanal , P. Ramos‐Cabrer , B. Yagüe , L. Aguado , L. Bardia , S. Tosi , V. Gómez‐Vallejo , A. Martín , T. Patiño , E. Julián , J. Colombelli , J. Llop , S. Sánchez , Nat. Nanotechnol. 2024, 19, 554. 38225356 10.1038/s41565-023-01577-y PMC11026160

[8] A. Abramson , E. Caffarel‐Salvador , M. Khang , D. Dellal , D. Silverstein , Y. Gao , M. R. Frederiksen , A. Vegge , F. Hubálek , J. J. Water , A. V. Friderichsen , J. Fels , R. K. Kirk , C. Cleveland , J. Collins , S. Tamang , A. Hayward , T. Landh , S. T. Buckley , N. Roxhed , U. Rahbek , R. Langer , G. Traverso , Science 2019, 363, 611. 30733413 10.1126/science.aau2277 PMC6430586

[9] S. Jeon , S. Kim , S. Ha , S. Lee , E. Kim , S. Y. Kim , S. H. Park , J. H. Jeon , S. W. Kim , C. Moon , B. J. Nelson , J.‐y. Kim , S.‐W. Yu , H. Choi , Sci. Rob. 2019, 4, eaav4317.

[10] I. S. M. Khalil , H. C. Dijkslag , L. Abelmann , S. Misra , Appl. Phys. Lett. 2014, 104, 223701.

[11] R. Dreyfus , J. Baudry , M. L. Roper , M. Fermigier , H. A. Stone , J. Bibette , Nature 2005, 437, 862. 16208366 10.1038/nature04090

[12] M. Roper , R. Dreyfus , J. Baudry , M. Fermigier , J. Bibette , H. A. Stone , Proceedings of the Royal Society A: Mathematical, Physical and Engineering Sciences 2008, 464, 877.

[13] T. Li , J. Li , K. I. Morozov , Z. Wu , T. Xu , I. Rozen , A. M. Leshansky , L. Li , J. Wang , Nano Lett. 2017, 17, 5092. 28677387 10.1021/acs.nanolett.7b02383

[14] H.‐W. Huang , M. S. Sakar , A. J. Petruska , S. Pané , B. J. Nelson , Nat. Commun. 2016, 7, 12263. 27447088 10.1038/ncomms12263 PMC5512624

[15] W. Hu , G. Z. Lum , M. Mastrangeli , M. Sitti , Nature 2018, 554, 81. 29364873 10.1038/nature25443

[16] Z. Ren , W. Hu , X. Dong , M. Sitti , Nat. Commun. 2019, 10, 2703. 31266939 10.1038/s41467-019-10549-7 PMC6606650

[17] V. Magdanz , J. Gebauer , D. Mahdi , J. Simmchen , I. S. Khalil , in Manipulation, Automation and Robotics at Small Scales (MARSS), International Conference on, IEEE, Helsinki, 2019.

[18] V. Magdanz , I. S. Khalil , J. Simmchen , G. P. Furtado , S. Mohanty , J. Gebauer , H. Xu , A. Klingner , A. Aziz , M. Medina‐Sánchez , O. G. Schmidt , S. Misra , Sci. Adv. 2020, 6, eaba5855. 32923590 10.1126/sciadv.aba5855 PMC7450605

[19] I. S. M. Khalil , A. F. Tabak , K. Sadek , D. Mahdy , N. Hamdi , M. Sitti , IEEE Robot. Autom. Lett. 2017, 2, 927.

[20] L. Yang , M. Zhang , H. Yang , Z. Yang , L. Zhang , IEEE Int. Conf. Intell. Robots Syst. 2021, 7476.

[21] L.‐J. W. Ligtenberg , N. C. A. Rabou , C. Goulas , W. C. Duinmeijer , F. R. Halfwerk , J. Arens , R. Lomme , V. Magdanz , A. Klingner , E. A. M. Klein Rot , C. H. E. Nijland , D. Wasserberg , H. R. Liefers , P. Jonkheijm , A. Susarrey‐Arce , M. Warlé , I. S. M. Khalil , Comms. Eng. 2024, 3, 68. 10.1038/s44172-024-00215-2 PMC11099159 39901022

[22] A. C. Bakenecker , A. von Gladiss , H. Schwenke , A. Behrends , T. Friedrich , K. Lüdtke‐Buzug , A. Neumann , J. Barkhausen , F. Wegner , T. M. Buzug , Sci. Rep. 2021, 11, 14082. 34234207 10.1038/s41598-021-93323-4 PMC8263782

[23] Q. Jin , Y. Yang , J. A. Jackson , C. Yoon , D. H. Gracias , Nano Lett. 2020, 20, 5383. 32463679 10.1021/acs.nanolett.0c01729 PMC7405256

[24] P. Shokrollahi , Y. P. Lai , S. Rash‐Ahmadi , V. Stewart , M. Mohammadigheisar , L. A. Huber , N. Matsuura , A. E. H. Zavodni , J. Parkinson , E. Diller , IEEE/ASME Trans. Mechatron. 2021, 26, 2616.

[25] C. Ridzewski , M. Li , B. Dong , V. Magdanz , ACS Appl. Bio Mater. 2020, 3, 1616. 10.1021/acsabm.9b01188 35021652

[26] V. Magdanz , M. Guix , F. Hebenstreit , O. G. Schmidt , Adv. Mater. 2016, 28, 4048. 10.1002/adma.201505487 27003908

[27] H. Xu , M. Medina‐Sánchez , V. Magdanz , L. Schwarz , F. Hebenstreit , O. G. Schmidt , ACS Nano 2017, 12, 327. 29202221 10.1021/acsnano.7b06398

[28] M. Dong , X. Wang , X.‐Z. Chen , F. Mushtaq , S. Deng , C. Zhu , H. Torlakcik , A. Terzopoulou , X.‐H. Qin , X. Xiao , J. Puigmartí‐Luis , H. Choi , A. P. Pêgo , Q.‐D. Shen , B. J. Nelson , S. Pané , Adv. Funct. Mater. 2020, 30, 1910323.

[29] C. Thongprayoon , A. E. Krambeck , A. D. Rule , Nat. Rev. Nephrol. 2020, 16, 736. 32753740 10.1038/s41581-020-0320-7

[30] F. Grases , A. I. Villacampa , A. Costa‐Bauzá , O. Söhnel , Clin. Chim. Acta 2000, 302, 89. 11074067 10.1016/s0009-8981(00)00359-4

[31] F. Julià , A. Costa‐Bauza , F. Berga , F. Grases , World J. Urol. 2022, 40, 2105. 35689678 10.1007/s00345-022-04059-3 PMC9279199

[32] S. P. Dretler , R. C. Pfister , Annu. Rev. Med. 1983, 34, 359. 6344765 10.1146/annurev.me.34.020183.002043

[33] I. E. E. H. Hans‐Göran Tiselius Annika Andersson , Ann Borrud‐Ohlsson , Scand. J. Urol. Nephrol. 1999, 33, 286. 10572989

[34] A. Diri , B. Diri , Renal Failure 2018, 40, 357. 29658394 10.1080/0886022X.2018.1459306 PMC6014528

[35] L. Tzelves , P. Mourmouris , A. Skolarikos , Curr. Opin. Urol. 2021, 31, 102. 33332876 10.1097/MOU.0000000000000844

[36] A. Khabbazian , M. B. Khamesee , V. Magdanz , in International Conference on Manipulation, Automation and Robotics at Small Scales (MARSS), IEEE, Delft, 2024.

[37] A. Tsaturyan , E. Bokova , P. Bosshard , O. Bonny , D. G. Fuster , B. Roth , Urolithiasis 2020, 48, 501. 32770255 10.1007/s00240-020-01204-8 PMC7666279

[38] A. Ong , G. Brown , T. Tokas , B. M. Hameed , J. Philip , B. K. Somani , Curr. Urol. Rep. 2023, 24, 355. 37079196 10.1007/s11934-023-01164-7

[39] S. R. Khan , M. S. Pearle , W. G. Robertson , G. Gambaro , B. K. Canales , S. Doizi , O. Traxer , H. G. Tiselius , Nat. Rev. Dis. Primers 2016, 2. 10.1038/nrdp.2016.8 PMC5685519 27188687

[40] N. Sarigul , F. Korkmaz , l. Kurultak , Sci. Rep. 2019, 9, 20159. 31882896 10.1038/s41598-019-56693-4 PMC6934465

[41] A. J. Y. Chee , C. K. Ho , B. Y. S. Yiu , A. C. H. Yu , IEEE Trans Ultrasonics, Ferroelectrics, and Frequency Control Society 2016, 63, 1852. 10.1109/TUFFC.2016.2591946 27429436

[42] R. Mestre . (2022). rafamestre/NMTT‐nanomicromotor‐tracking‐tool: NMTT v1.0.0 (v1.0.0). Zenodo, 10.5281/zenodo.5905482.

