# Supplementary material for: Kidney Stone Dissolution By Tetherless, Enzyme‐Loaded, Soft Magnetic Miniature Robots
Source: Adv Healthc Mater. 2025 Jul 1;14(23):2403423. doi: 10.1002/adhm.202403423 (PMC12417750; doi:10.1002/adhm.202403423)
Supplement: Supplementary file 1 — Supporting Information [file ADHM-14-0-s003.pdf]

ADVANCED HEALTHCARE MATERIALS

Supporting Information

for *Adv. Healthcare Mater.*, DOI 10.1002/adhm.202403423

Kidney Stone Dissolution By Tetherless, Enzyme-Loaded, Soft Magnetic Miniature Robots

*Afarin Khabbazian, Lauren Kwong, Aaron Lewis, Erica Liu, Noura Abdelrazec, Anna C. Bakenecker, Nil Fontanals, Guillem Lopez, Samuel Sánchez, Juan Manuel Lopez, Brian Carrillo, Monica Farcas, Chris Kallweit, Alfred C. H. Yu, Mir Behrad Khamesee and Veronika Magdanz**

SUPPORTING INFORMATION

Kidney Stone Dissolution by Tetherless, Enzyme-loaded, Soft Magnetic Miniature Robots

Afarin Khabbazian[1], *Lauren Kwong*[2], *Aaron Lewis*[2], *Erica Liu*[2], *Noura Abdelrazec*[2], *Anna C. Bakenecker*[3,4], *Nil Fontanals*[3], *Guillem Lopez*[3], *Samuel Sanchez*[3,5], *Juan Manuel Lopez*[6], *Brian Carrillo*[7], *Monica Farcas*[7], *Chris Kallweit*[8], *Alfred C. H. Yu*[8,9], *Mir Behrad Khamesee*[1], *Veronika Magdanz*[2,9]*

[1] Maglev Lab, Department of Mechanical Engineering, University of Waterloo, N2L 3G1 Waterloo, Canada,
[2] Medical Microrobotics Lab, Department of Systems Design Engineering, University of Waterloo, N2L 3G1 Waterloo, Canada,
[3] Institute for Bioengineering of Catalonia, 08028 Barcelona, Spain,
[4] Medical Engineering, Technical University of Darmstadt, Merckstr. 25, 64283 Darmstadt, Germany,
[5] Catalan Institute for Research and Advanced Studies, ICREA, 08010 Barcelona, Spain,
[6] Department of Urology, University of Barcelona Clinic Hospital, 08036 Barcelona, Spain,
[7] Farcas Lab, Department of Surgery, Division of Urology, St. Michael's Hospital, University of Toronto, M5B 1W8 Toronto, Canada
[8] Schlegel Research Institute for Aging and Department of Electrical and Computer Engineering, University of Waterloo, N2L 3G1 Waterloo, Canada
[9] Waterloo Institute for Nanotechnology, N2L 3G1 Waterloo, Canada
Email address: veronika.magdanz@uwaterloo.ca

1 Supporting Videos

Supporting Video 1: Magnetic flexible robot in the finbot configuration moving through the ureter model with 8Hz actuation frequency.
Supporting Video 2: Magnetic flexible robot in the screwbot configuration moving through the ureter model with 8Hz actuation frequency.
Supporting Video 3: Magnetic flexible robot in the finbot configuration moving through the bladder model with 2Hz actuation frequency.
Supporting Video 4: Magnetic flexible robot in the screwbot configuration moving through the bladder model with 2Hz actuation frequency.
Supporting Video 5: Magnetic flexible robot in the finbot configuration moving through the renal pelvis model with 8Hz actuation frequency.
Supporting Video 6: Magnetic flexible robot in the screwbot configuration moving through the renal pelvis model with 2Hz actuation frequency.
Supporting Video 7: Ultrasound imaging of the screwbot inside the bladder. The schematic indicates the field of view of the ultrasound image and location of robot inside the bladder. The robot rotations inside the bladder cause the switching between blue and red in the Doppler mode due to the fluid flow.
Supporting Video 8: Ultrasound imaging of the screwbot inside the renal pelvis with simultaneous magnetic actuation.
Supporting Video 9: Ultrasound imaging of the screwbot inside the ureter with simultaneous magnetic actuation video.

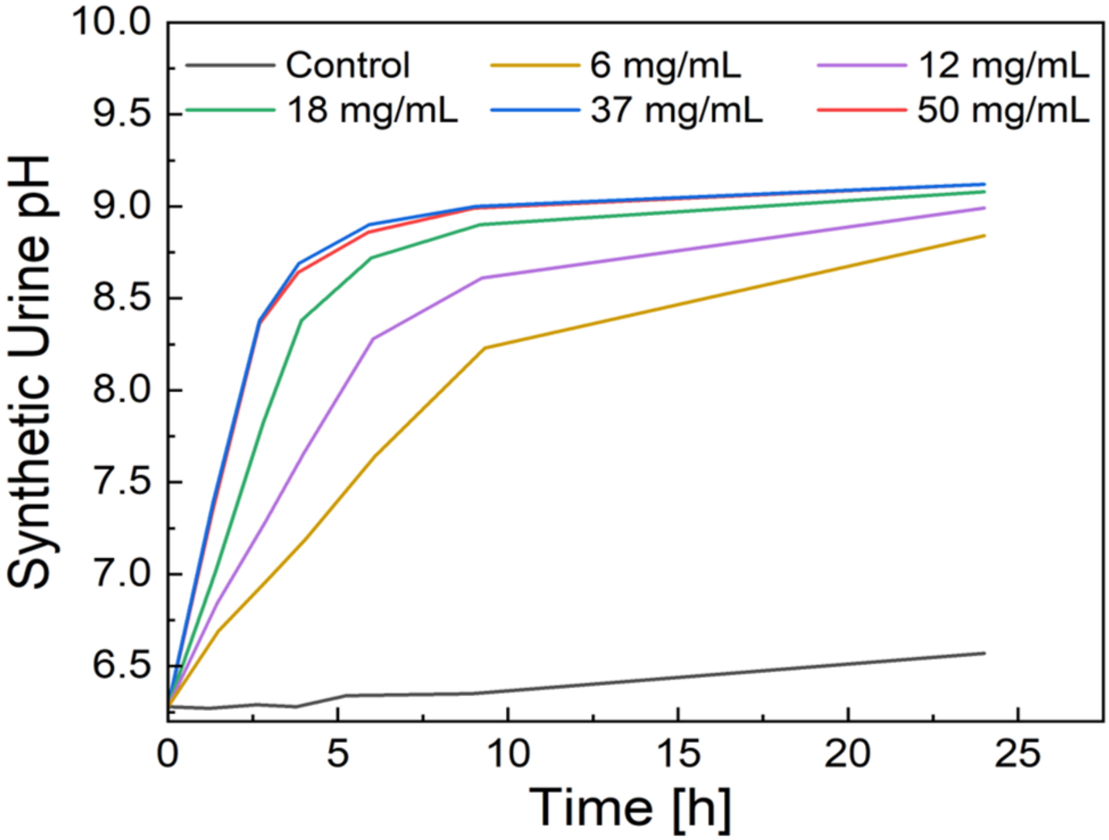

Figure S1: Synthetic urinary pH increase over 24 hours by GelMa robots with different embedded urease concentrations ranging from 0-50 mg/mL. The control refers to a GelMa robot without urease.

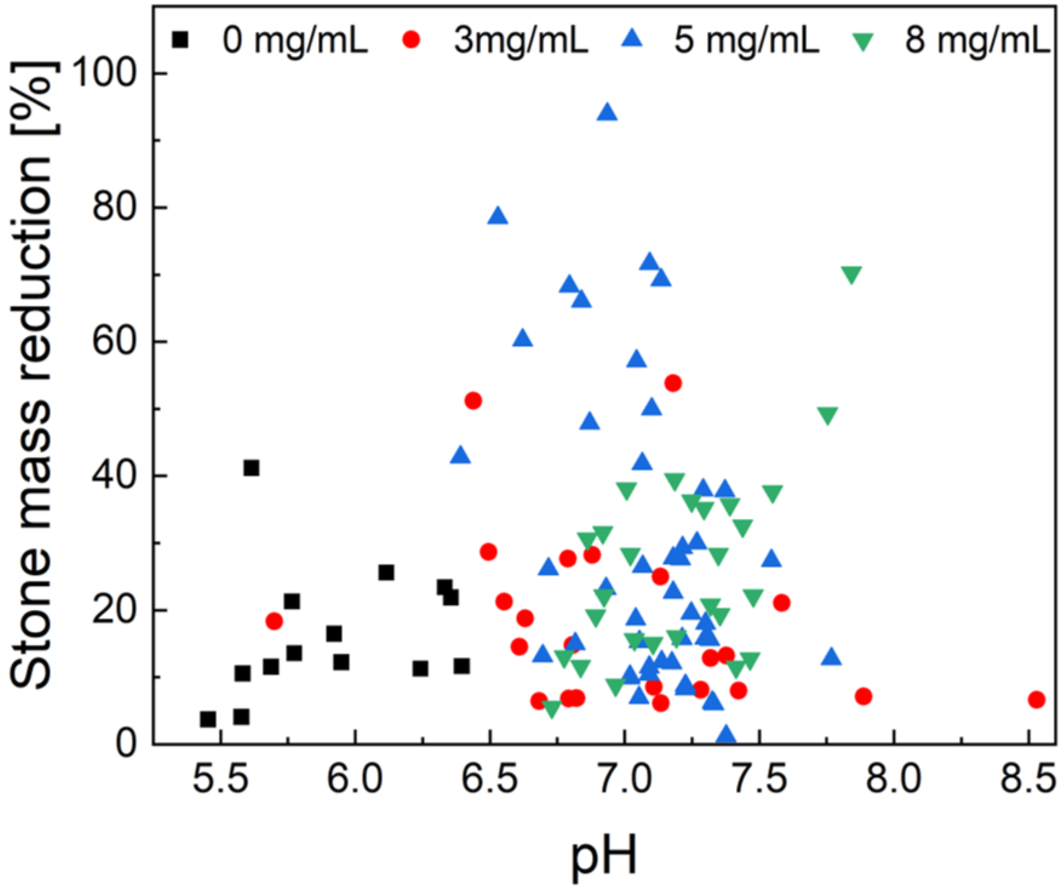

Figure S2: Scatter plot of kidney stone mass reduction in correlation to urinary pH increase. pH increase by GelMa robots with different embedded urease concentrations ranging from 0-8 mg/mL, with pH value obtained in synthetic artificial urine after 24 hours exposure.

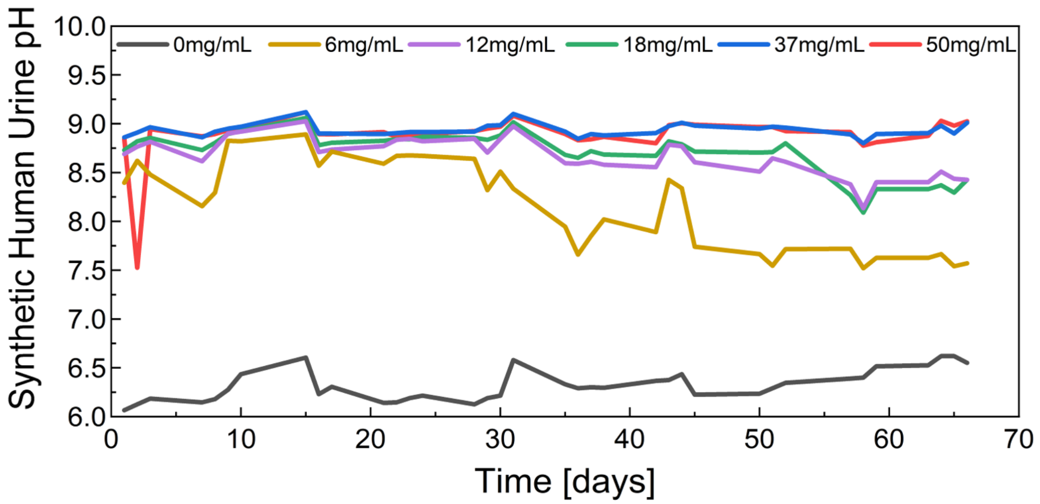

Figure S3: Prolonged urine pH increase by urease-loaded filaments. Every 24h, the solution is replaced with fresh synthetic urine of pH 6, and pH change is measured after 24h, indicating a consistent long-term pH increase over a period of 65 days. The lowest urease concentration of 6 mg/mL was sufficient for continuous pH increase.

Urease assay		Statistics					
Sample	Time point	Average urease activity [Units/L]	Standard error of the mean [Units/L]	Significant difference	R square	Coeff Var	Prob>F
1)Control	Day 0	14.96	2.16	***	0.99991	0.01199	<0.0001
2)3 mg/mL filament		240	2.71	n.s.	0.32798	0.00793	0.42731
3) 5 mg/mL filament		245	0.42	-	-	-	-
4) 8 mg/mL filament		262	23.85	n.s.	0.33326	0.06639	0.42271
5 mg/mL free enzyme		317	2.54	***	0.99845	0.00655	0.0007737
1)Control	Day 1	13	3.77	***	0.9963	0.07277	<0.0001
2)3 mg/mL filament		148	45.39	n.s.	0.50824	0.17259	0.07215
3) 5 mg/mL filament		246	12.42	-	-	-	-
4) 8 mg/mL filament		243	3.77	n.s.	0.7613	0.04362	0.05358
5 mg/mL free enzyme		259	3.2	-	-	-	-
1)Control	Day 10	3	1.86	**	0.99954	0.04917	0.01367
2)3 mg/mL filament		99	56	n.s.	0.41867	0.60901	0.35295
3) 5 mg/mL filament		90	52	-	-	-	-
4) 8 mg/mL filament		67	4.3	n.s.	0.1047	0.78024	0.67643
5 mg/mL free enzyme		109	1.19	n.s.	0.57574	0.35481	0.08026

Figure S4: Statistical Analysis performed by one-way ANOVA in Origin 2023b software

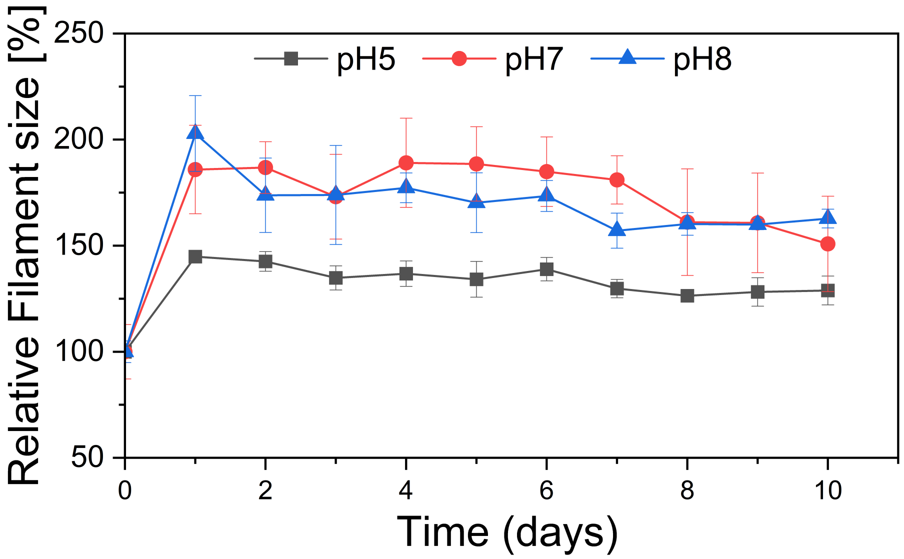

Figure S5: Swelling and degradation of GelMa filaments over 10 days. The relative filament size was measured by the area of the filaments in images taken daily. Day 0 corresponds to the filaments immediately after fabrication before immersing them in urine. Sample size is n=3 filaments per condition. Average data with standard error of the mean are displayed.
